# Assessing Discordance Between Self-Perceived HIV Risk and Laboratory-Confirmed HIV Status in South Africa: Insights from the 2022 National Population-Based Survey

**DOI:** 10.3390/ijerph23081083

**Published:** 2026-08-20

**Authors:** Sbonelo Chamane, Inbarani Naidoo, Musawenkosi Mabaso, Angeline Sibongile Ngcobo, Prudence Chambale, Tebogo Sole-Moloto, Lehlogonolo Makola, Sean Jooste, Lesiba Molopa, Thembelihle Ginyana, Sizulu Moyo, Nompumelelo Zungu, Khangelani Zuma

**Affiliations:** 1 Public Health, Societies and Belonging, Human Sciences Research Council, Pretoria 0038, South Africa; inaidoo@hsrc.ac.za (I.N.); mmabaso@hsrc.ac.za (M.M.); pechambale@hsrc.ac.za (P.C.); soletb@unisa.ac.za (T.S.-M.); lmakola@hsrc.ac.za (L.M.); sjooste@hsrc.ac.za (S.J.); lomolopa@hsrc.ac.za (L.M.); tginyana@hsrc.ac.za (T.G.); smoyo@hsrc.ac.za (S.M.); mzungu@hsrc.ac.za (N.Z.); kzuma@hsrc.ac.za (K.Z.); 2 School of Public Health, University of the Witwatersrand, Johannesburg 2193, South Africa; 3 Information and Corporate Management, Durban University of Technology, Durban 4001, South Africa; angelinen@dut.ac.za; 4 School of Public Health and Family Medicine, University of Cape Town, Cape Town 7925, South Africa; 5 School of Nursing and Public Health, University of KwaZulu-Natal, Durban 4041, South Africa

**Keywords:** HIV, self-perceived risk, discordance, risk of HIV

## Abstract

**Highlights:**

**Public health relevance—How does this work relate to a public health issue?**

- This study examined the discordance between self-perceived HIV risk and laboratory-confirmed HIV status using nationally representative data from South Africa.
- The findings address an important public health challenge, as inaccurate HIV risk perception may influence HIV testing uptake, prevention behaviours, and timely initiation of treatment.

**Public health significance—Why is this work of significance to public health?**

- Approximately 4.1% of participants tested HIV positive despite perceiving themselves to be at low risk of HIV infection, while 11.5% perceived themselves to be at high risk but tested HIV negative. Additionally, 1.0% of participants self-reported being HIV positive despite laboratory-confirmed results indicating they were HIV negative.
- HIV risk perception discordance was more common among young and middle-aged adults, Black Africans, females, and residents of rural areas, highlighting important demographic inequalities in HIV awareness and vulnerability.

**Public health implications—What are the key implications or messages for practitioners, policy makers and/or researchers in public health?**

- HIV prevention programmes should strengthen accurate HIV risk perception through targeted and culturally responsive health communication interventions.
- Public health strategies should prioritise populations with higher levels of discordance to improve HIV testing, prevention uptake, and early linkage to HIV care and treatment.

**Abstract:**

Background: The discordance between individuals’ self-perceived risk of HIV infection and laboratory-confirmed risk has a significant impact on behaviours related to HIV prevention and timely initiation of treatment. Methods: Data was obtained from the sixth South African National HIV Prevalence, Incidence, and Behaviour Survey. Descriptive statistics were used to summarise the sample characteristics and provide the prevalence of discordance. A multivariable multinomial logistic regression was employed to investigate factors associated with discordant results. Results: Of 34,528 participants, 4.1% (95% Confidence Interval (CI): 3.7–4.5) were classified as HIV-positive with low perceived risk, while 11.5% (95% CI: 10.4–12.6) were classified as HIV-negative with high perceived risk and 1.0% (95% CI: 0.8–1.1) as self-misdiagnosed. Being HIV-positive with low perceived risk was significant among older ages (25–54 years) and lower among other races than Black Africans and those residing in rural informal areas. In contrast, being HIV-negative with high perceived risk of HIV risk was more common among participants aged 25–44 years, as well as those living in rural informal and rural formal areas, but lower among other races than Black Africans. The factor associated with self-misdiagnosis was being female. However, participants aged 15–24 years who were never married were less likely to self-misdiagnose. Conclusion: These findings highlight a significant discrepancy between perceived and laboratory-confirmed HIV risk, underscoring the importance of targeted interventions to improve risk perception accuracy. Enhancing individuals’ understanding of their true risk may lead to better engagement in preventive measures and the timely initiation of treatment.

## 1. Introduction

Self-perceived risk of HIV is one of the key drivers influencing whether individuals adopt behaviours that either reduce or heighten their risk of contracting sexually transmitted infections, including HIV. However, evidence continues to show that often people inaccurately assess their actual level of risk of acquiring HIV, leading to discordance between self-perceived HIV risk and their laboratory-confirmed HIV status [1,2,3,4]. Subsequently, these inaccurate self-perceived risks may hinder access to care and lower protective behavioural practices, such as consistent condom use, testing for HIV, and pre-exposure prophylaxis uptake [5,6,7]. Ultimately, the consequences of discordant practices may impede meeting the UNAIDS 95-95-95 targets by 2025. This is particularly concerning for countries such as South Africa, where, despite a decline in HIV prevalence as of 2022, over 7 million people are estimated to live with HIV, among whom a proportion remain unaware of their HIV-positive status and are virally unsuppressed [8].

Literature shows that an individual’s self-risk perception is closely associated with their proclivity to engage in risky sexual behaviours, which are a common mode of HIV transmission in South Africa [5,9]. Pringle used the Health Belief Model to argue that an individual’s health-seeking behaviour is influenced by the health education available to them [10], thus allowing for informed decision-making regarding the use of HIV prevention interventions. This level of autonomy has been valued and respected; however, research shows that it can lead to cases of risk perception bias or discordance, whereby individuals misjudge their likelihood of contracting or transmitting HIV [11]. This has been documented to occur in three ways: underestimation of risk, overestimation of protection and optimism bias [12].

Discordance between self-perceived levels of HIV risk and risky sexual behaviour has been previously suggested to occur among those with limited HIV knowledge, unaware of their HIV status or partner’s HIV status, those who engaged in behaviours that heightened the risk of HIV infection, had early sexual debut and those who hold stigmatising attitudes towards people living with HIV [4,13,14,15]. Although studies have been conducted across diverse population groups, there is limited nationally representative data assessing the discordance between self-perceived HIV risk and laboratory-confirmed HIV status in South Africa. In light of this gap, this paper examines the prevalence of, and sociodemographic factors associated with, risk perception discordance (HIV-positive with low perceived risk, HIV-negative with high perceived risk, self-misdiagnosed) using the most recent nationally representative data from the sixth South African National HIV Prevalence, Incidence, Behaviour and Communication Survey.

## 2. Materials and Methods

### 2.1. Study Population and Data

Data were obtained from the sixth South African National HIV Prevalence, Incidence, and Behaviour Survey. A detailed outline of the methods is available elsewhere [16]. Briefly, this cross-sectional, population-based household survey used a two-stage stratified cluster random sampling design. A systematic random sample of 1000 small area layers (SALs) was drawn from Statistics South Africa’s master sample, with 15 visiting points (VPs) and households sampled per SAL, targeting 15,000 VPs/households. An additional 1144 SALs were selected in 33 of the 52 districts to provide district-level estimates, yielding a total of 2144 SALs, stratified by province and locality type (urban, rural formal, and rural informal). All individuals in selected households were eligible to participate. A household questionnaire was administered to household heads, and age-appropriate individual behavioural questionnaires were given to consenting/assenting participants. The survey collected data on HIV-related knowledge, attitudes, behaviours, ART use, and adherence to treatment. Dried blood spots (DBSs) were collected for HIV testing, assessing HIV sero-status, incidence, antiretroviral drug presence, viral load, and HIV drug resistance. Data collection was conducted between January 2022 and April 2023 across all nine provinces of South Africa. Data were collected electronically using tablet-based questionnaires, comprising a household questionnaire and age-specific individual behavioural questionnaires designed for parents or guardians of children aged 0–11 years, children aged 12–14 years, and participants aged 15 years and older. Questions assessing self-perceived HIV risk were included only in the individual behavioural questionnaire administered to participants aged 15 years and older. For this study, the analysis was restricted to participants who were 15 years and older and had no missing information on self-perceived status and laboratory-confirmed HIV status.

### 2.2. Objectives

This study aimed to assess the discordance between self-perceived HIV risk and laboratory-confirmed HIV status among individuals aged 15 years and older in South Africa using nationally representative data from the sixth South African National HIV Prevalence, Incidence, Behaviour and Communication Survey.

Specifically, the study sought to:

- Estimate the prevalence of concordance and discordance between self-perceived HIV risk and laboratory-confirmed HIV status, including HIV-positive individuals with low perceived risk, HIV-negative individuals with high perceived risk, and individuals who self-reported being HIV-positive but tested HIV-negative.
- Determine the sociodemographic and socioeconomic factors associated with each category of HIV risk-perception discordance, using the concordant group as the reference category.

### 2.3. Measures

#### 2.3.1. Dependent Variable

Participants were asked to rate their self-perceived risk of acquiring HIV using a scale from 1 to 4 (1 = You definitely will not get infected with HIV, 2 = You probably will not get infected, 3 = You are probably going to get infected, and 4 = You are definitely going to get infected). An additional response option (coded as 9) was included for those who reported being HIV positive. For the analysis, responses 1 and 2 were grouped together to represent low perceived risk, while responses 3 and 4 were grouped to represent high perceived risk. Individuals who selected option 9 (already HIV positive) were kept as a separate category, as they were not at risk of future acquisition and could not meaningfully assess the risk of becoming infected. This question was administered before HIV testing during data collection, such that participants’ perceived risk of future HIV acquisition was assessed prior to determination of their laboratory-confirmed HIV status.

Laboratory-confirmed HIV status was recorded separately and coded as either positive or negative. HIV testing was subsequently conducted following the assessment of self-perceived HIV risk. These two variables (self-perceived HIV risk and laboratory-confirmed HIV status) were used to construct a new outcome variable capturing risk perception discordance. The comparison between self-perceived future HIV acquisition risk and subsequently determined laboratory-confirmed HIV status was therefore used to assess whether participants’ prospective perceptions of their HIV risk were consistent with their objectively measured HIV status.

The Risk Perception Discordance variable was used as the dependent variable and categorised into four mutually exclusive groups based on the cross-classification of self-perceived HIV risk and laboratory-confirmed HIV status. Concordant (0) included participants with low perceived risk (responses 1–2) who tested HIV-negative, those with high perceived risk (responses 3–4) who tested HIV-positive, and participants who reported being HIV-positive (response 9) and were confirmed HIV-positive by laboratory testing. HIV-positive with low perceived risk (1) included participants with low perceived risk (responses 1–2) who tested HIV-positive, while HIV-negative with high perceived risk (2) included participants with high perceived risk (responses 3–4) who tested HIV-negative. Self-misdiagnosed (3) included participants who selected response 9, indicating that they considered themselves HIV-positive, but whose laboratory test result was HIV-negative (see Table 1).

#### 2.3.2. Independent Variables

The socio-demographic variables explored in this study were age groups (15–24, 25–34, 35–44, 45–54, 55–64, and 65+ years), individual sex (male, female), race groups (Black African, other races), marital status (married, never married, divorced/widowed), highest education level (none/primary school, some secondary school/Grade 12, tertiary education), employment status (unemployed, employed), locality type (urban areas, rural informal tribal areas, rural formal farm areas) and asset-based social economic status (SES) (low SES, high SES), constructed using multiple correspondence analyses based on a series of indicators of household assets and access to utilities from the survey’s household questionnaire.

### 2.4. Statistical Analysis

The statistical analyses were performed using Stata statistical software version 18.0 (Stata Corporation, College Station, TX, USA). All analyses accounted for the complex survey design of SABSSM VI by incorporating the survey weights, sampling strata, and primary sampling units (PSUs) in the variance estimation. Descriptive statistics were used to summarise the sample characteristics and provide the prevalence of discordance measured using frequencies and percentages. A bivariate multinomial logistic regression was employed to investigate sociodemographic and socioeconomic factors associated with discordant results. Factors that were significant in the bivariate multinomial logistic regression were used to fit a multivariable multinomial logistic regression. The multivariable analyses were conducted using a complete-case approach, with participants with missing data on any variables included in the respective model excluded from the analysis. The proportion of missing data was no more than 5%; therefore, further investigation of missingness or imputation was not considered necessary. For both models, the concordance group was used as the baseline for the multinomial estimation. The adjusted relative risk ratio (aRRR) and 95% confidence interval (CI) were used to measure the direction and strength of the association at *p* ≤ 0.05, which reflects statistical significance.

## 3. Results

### 3.1. Sample Characteristics

The analytical sample consisted of 34,528 participants with complete data for risk perception concordance and discordance with the laboratory-confirmed HIV status (Table 2). The majority of participants tested HIV negative (83.7%), self-reported low risk perception of HIV (75.3%), were aged 25 to 34 years (24.8%), female (54.4%), Black African (78.6%), never married (63.5%), had completed some secondary school/Grade 12 (67.4%), were unemployed (68.6%), resided in urban areas (66.2%) and classified as high SES (53.4%).

### 3.2. Prevalence of Discordance

Of 34,528 participants, 4.1% (95% CI: 3.7–4.5) were classified as HIV-positive with low perceived risk, while 11.5% (95% CI: 10.4–12.6) were HIV-negative with high perceived risk and 1.0% (95% CI: 0.8–1.1) were self-misdiagnosed (Table 3). A higher prevalence of participants who were HIV-positive with low perceived risk was among participants aged 35 to 44 years, among females, Black Africans, those who were never married, those who completed none/primary school, were employed, resided in rural formal areas and classified as low SES. Participants aged 25 to 34 years, males, Black Africans, those who were never married, those who completed some secondary school/Grade 12, unemployed, resided in rural informal areas, and classified as low SES had a higher prevalence of HIV-negative with high perceived risk. Moreover, self-misdiagnosis was higher among participants aged 45 to 54 years, females, Black Africans, those who were never married, those who completed none/primary school, unemployed, resided in rural informal areas, and classified as low SES.

### 3.3. Bivariate Multinomial Logistic Regression

The bivariate multinomial logistic regression was employed to investigate sociodemographic and socioeconomic factors associated with the multilevel discordant variable. Factors significant in the bivariate analysis were used to fit a multivariable multinomial logistic regression. Almost all sociodemographic and socioeconomic factors showed significant associations with discordance, except for employment status (Table 4).

### 3.4. Multivariable Multinomial Logistic Regression

Table 5 presents the results of the multivariable multinomial logistic regression of socio-demographic factors associated with HIV risk perception discordance, using the concordant group as the reference category. Of the 34,528 participants, 32,790 (95.0%) had complete information and were included in the final multivariable analysis, while 1738 (5.0%) were excluded because of missing data. Participants aged 25–34 years, 35–44 years, and 45–54 years had higher relative risks of being in the HIV-positive with low perceived risk group compared to the concordant group than participants aged 60 years and older (aRRR = 2.13; 95% CI: 1.27–3.55, aRRR = 3.07; 95% CI: 1.85–5.10, and aRRR = 2.79; 95% CI: 1.70–4.60, respectively). In contrast, participants from other race groups were less likely than Black Africans to be in the HIV-positive with low perceived risk group relative to the concordant group (aRRR = 0.18; 95% CI: 0.10–0.32). Similarly, residents of rural informal areas were less likely than those in urban areas to be in the HIV-positive with low perceived risk group compared to the concordant group (aRRR = 0.70; 95% CI: 0.53–0.92).

Participants aged 25–34 and 35–44 years had higher adjusted relative risks of being in the HIV-negative with high perceived risk group compared to the concordant group than those aged 60 years and older (aRRR = 3.32; 95% CI: 1.43–7.73 and aRRR = 2.52; 95% CI: 1.07–5.91, respectively). Similarly, participants living in rural informal and rural formal areas were more likely than urban residents to be HIV-negative with high perceived risk relative to the concordant group (aRRR = 2.01; 95% CI: 1.53–2.65 and aRRR = 1.91; 95% CI: 1.26–2.89, respectively). In contrast, participants from other race groups were less likely than Black Africans to be HIV-negative with high perceived risk compared to the concordant group (aRRR = 0.47; 95% CI: 0.30–0.74).

Being female was associated with a higher adjusted relative risk of self-misdiagnosis compared to males (aRRR = 1.99; 95% CI: 1.32–2.99) in relation to the concordant group. Conversely, participants aged 15–24 years were less likely than those aged 60 years and older to self-misdiagnose (aRRR = 0.11; 95% CI: 0.04–0.35) compared to the concordant group. Additionally, never-married participants were more likely than married participants to be in the self-misdiagnosed group relative to the concordant group (aRRR = 2.23; 95% CI: 1.44–3.47).

## 4. Discussion

The study aimed to examine the prevalence and sociodemographic factors associated with HIV risk perception discordance. The findings of this study highlight the prevalence of discordance between self-perceived HIV risk and laboratory-confirmed HIV status, with 4.1% of participants classified as HIV-positive with low perceived risk of HIV infection, while 11.5% were HIV-negative with high perceived risk and 1.0% were self-misdiagnosed. Higher levels of discordance were noted among demographic groups with higher levels of HIV prevalence in South Africa, this includes young adult and middle-aged individuals compared to participants 65 years and older, females compared to males [17,18], Black Africans compared to other race groups [19], never married compared to married [20,21], lower levels of education compared to tertiary education [22,23,24], unemployed compared to employed [25], and low SES compared to high SES [26,27]. However, residing in rural formal and informal compared to urban areas was the exception to this observation between HIV prevalence and discordance results [18,28]. These results highlight a connection between discordance and prevalence and suggest that risk perception discordance may play a crucial role in driving the HIV epidemic in South Africa.

The finding reveals that young adult individuals were more likely to be both HIV-positive with low perceived risk and HIV-negative with high perceived risk compared to those 65 years and older. However, the youth and adolescents were less likely to self-misdiagnose. Young adults generally perceive a lower level of personal risk when engaging in potentially dangerous activities, often attributed to a sense of invulnerability associated with adolescence, meaning they may underestimate the potential negative consequences of risky behaviours compared to adults [29,30,31]. Studies have shown that youth engage in risky sexual behaviours such as multiple sexual partnerships and no condom use without fully recognising the associated risks [32]. This could be due to a lack of comprehensive sex education, peer pressure, low perceived relevance of HIV to their social group, or trust in their partners without verification of HIV status.

This study further revealed that females were more likely to self-misdiagnose compared to males. However, females are disproportionately affected by HIV in South Africa [18,33], and some studies argue that women might have lower levels of HIV prevention knowledge [34]. Additionally, their limited ability to negotiate condom use and practice safe sex is a challenge [35,36]. Social and cultural factors play a crucial role in these disparities and may further contribute to this discordance. These findings underscore the importance of strengthening programs that offer sex education to women such as family planning to encompass social and cultural elements. Additionally, studies have shown that inclusion of a male sexual partner during family planning improves a female’s reproductive health [37].

The findings showed that Black Africans had a higher level of discordance, both HIV-positive with low perceived risk and HIV-negative with high perceived risk. Research has shown that African participants were significantly more likely to rate their risk as high (11.6%) compared to non-Africans (2.8%, *p* = 0.001) [38]. In South Africa, this population group has the highest prevalence of HIV compared to other racial groups [19]. This has been attributed to contextual and structural drivers faced by this group [39,40]. Given these findings, targeted interventions are needed to strengthen accurate risk perception, including culturally responsive health communication, tailored community-based education, and programs that directly address structural barriers such as poverty, stigma, and limited healthcare access.

Consistent with other studies [41], participants who were never married were more likely to self-misdiagnose. This could be due to fewer opportunities for discussions about HIV and sexual health within stable relationships, as well as lower perceived risk due to fewer long-term partners. Literature also suggests otherwise, pointing to married women and their ability to underestimate their risk of HIV because they perceive their partner as monogamous and their limited ability to negotiate safe sexual practices [42,43]. These contrasting findings suggest that interventions should not only target unmarried individuals but also address the specific vulnerabilities faced by married women, including empowering them in sexual health decision-making and fostering open communication within relationships.

The final model also revealed that residents of rural areas, both formal and informal, had a higher likelihood of being HIV-negative with high perceived risk, while urban areas were more likely to be HIV-positive with low perceived risk. Similar findings have been observed in other studies [44]. Rural area residents are one of the marginalised groups of people in South Africa [45], and research has shown that lower risk perception among marginalised populations results in an increase in HIV incidence [2,46,47].

### Strengths & Limitations

The study’s nature limits the ability to infer causal relationships. The study used some self-reported variables, such as self-perceived risk of HIV, which may be subject to social desirability bias. Although perceived HIV acquisition risk was assessed before HIV testing, it represents a distinct construct from laboratory-confirmed HIV status; therefore, discordance should be interpreted as a mismatch between prospective perceived risk and subsequently determined HIV status rather than as a direct measure of future HIV acquisition risk. Another limitation of this study is that Coloured, Indian/Asian, and White participants were combined into an “Other” category due to small sample sizes, which may mask important differences among these population groups. Despite these limitations, a key strength of this study is the use of nationally representative data from SABSSM VI, which provides broad population coverage across all nine provinces of South Africa. The large sample size and use of laboratory-confirmed HIV status alongside self-reported risk perception also strengthen the validity and generalisability of the findings. Furthermore, accounting for the complex survey design and sampling weights enhances the representativeness of the estimates.

## 5. Conclusions

This study assessed the prevalence and sociodemographic factors associated with discordance between self-perceived HIV risk and laboratory-confirmed HIV status among South Africans aged 15 years and older. We found that 4.1% of participants were HIV-positive despite reporting low perceived risk, 11.5% were HIV-negative despite reporting high perceived risk, and 1.0% self-reported being HIV-positive despite testing HIV-negative. Descriptively, the prevalence of risk perception discordance varied across age, sex, race, marital status, education, socioeconomic status, and locality type. However, after adjustment for other sociodemographic and socioeconomic factors, only selected characteristics remained independently associated with the different forms of discordance. These findings demonstrate that both underestimation and overestimation of HIV risk remain important public health concerns in South Africa. HIV prevention and testing programmes should strengthen accurate HIV risk assessment through targeted, culturally responsive, and context-specific health communication, particularly among populations experiencing greater discordance. Future research should examine the underlying behavioural, social, and structural mechanisms contributing to discordance and assess whether interventions designed to improve HIV risk perception can increase HIV testing, prevention uptake, and timely linkage to care.

## Figures and Tables

**Table 1:** Classification of HIV risk-perception concordance and discordance according to self-perceived HIV risk and laboratory-confirmed HIV status.

Self-Perceived HIV Risk	Laboratory-Confirmed HIV Status	Classification
Low perceived risk (1–2)	HIV-negative	Concordant
High perceived risk (3–4)	HIV-positive	Concordant
Reported HIV-positive (9)	HIV-positive	Concordant
Low perceived risk (1–2)	HIV-positive	HIV-positive with low perceived risk
High perceived risk (3–4)	HIV-negative	HIV-negative with high perceived risk
Reported HIV-positive (9)	HIV-negative	Self-misdiagnosed

**Table 2:** Socio-demographic characteristics of participants with self-perceived risk of acquiring HIV and laboratory-confirmed HIV status, South Africa, 2022.

Variables	*n*	Column %	95% CI
Total	34,528	100.0	
Final HIV Status			
Non-Reactive	27,234	83.7	82.8–84.6
Reactive	7294	16.3	15.4–17.2
Self–perceived risk of HIV			
Low risk	24,784	75.3	74.0–76.7
High risk	4222	12.9	11.8–14.1
HIV Positive	5522	11.8	11.0–12.6
Age groups (years)			
15–24	8828	22.5	21.5–23.5
25–34	7586	24.8	23.5–26.1
35–44	6039	20.6	19.6–21.7
45–54	4500	13.2	12.3–14.1
55–64	3935	10.1	9.4–10.9
65+	3640	8.8	8.0–9.6
Sex			
Male	13,572	45.6	44.3–46.8
Female	20,938	54.4	53.2–55.7
Race groups			
Black African	31,236	78.6	75.8–81.1
Other ^1^	3254	21.4	18.9–24.2
Marital status			
Ever Married	10,439	36.5	34.6–38.5
Never Married	23,931	63.5	61.5–65.4
Highest education level			
None/Primary school	7783	16.9	15.8–18.0
Some Secondary school/Grade 12	22,969	67.4	65.9–68.9
Tertiary education	3473	15.7	14.1–17.4
Employment status			
Unemployed	24,894	68.6	66.9–70.3
Employed	8897	31.4	29.7–33.1
Locality type			
Urban areas	20,432	66.2	62.9–69.4
Rural informal (tribal areas)	9704	26.9	23.9–30.1
Rural formal (farm areas)	4392	6.8	5.5–8.5
Asset-based SES			
Low SES	18,454	46.6	43.3–50.0
High SES	14,740	53.4	50.0–56.7

^1^ Other includes White, Asian and Coloured population groups. The frequencies presented for individual variables do not necessarily sum to the total sample size (*N* = 34,528) because of missing values. The reported frequencies are based on the available observations for each variable.

**Table 3:** Prevalence of concordance and discordance for HIV risk perception and laboratory-confirmed HIV status among participants aged 15 years and older, South Africa, 2022.

Variables	Sample	Concordant		HIV-Positive with Low Perceived Risk		HIV-Negative with High Perceived Risk		Self-Misdiagnosed		*p*-Value ^1^
	*n*	Row %	95% CI	Row %	95% CI	Row %	95% CI	Row %	95% CI	
Total	34,528	83.5	82.3–84.6	4.1	3.7–4.5	11.5	10.4–12.6	1.0	0.8–1.1	
Age groups (years)										<0.001
15–24	8828	84.5	82.8–86.1	2.6	2.0–3.2	12.7	11.1–14.4	0.2	0.1–0.4	
25–34	7586	77.8	75.2–80.2	4.5	3.8–5.4	16.5	14.2–19.1	1.2	0.9–1.7	
35–44	6039	81.2	79.0–83.3	5.9	4.8–7.3	11.7	10.0–13.6	1.1	0.9–1.5	
45–54	4500	85.1	82.9–87.0	5.5	4.3–6.8	7.9	6.6–9.6	1.5	1.1–2.2	
55–64	3935	89.7	87.9–91.3	2.9	2.3–3.7	6.8	5.4–8.4	0.6	0.4–1.0	
65+	3640	92.5	89.7–94.6	1.7	1.2–2.4	4.6	2.8–7.5	1.2	0.7–2.0	
Sex										0.002
Male	13,572	83.4	81.8–84.9	3.8	3.2–4.4	12.2	10.8–13.8	0.6	0.4–0.8	
Female	20,938	83.5	82.1–84.9	4.3	3.8–5.0	10.9	9.7–12.2	1.3	1.0–1.5	
Race groups										<0.001
Black African	31,236	80.5	79.1–81.9	4.9	4.4–5.4	13.5	12.2–14.8	1.1	1.0–1.4	
Other	3254	94.3	92.4–95.7	1.1	0.7–1.7	4.4	3.1–6.2	0.3	0.1–0.6	
Marital status										<0.001
Ever Married	10,439	88.5	87.0–89.8	3.1	2.5–3.7	7.7	6.5–9.0	0.8	0.6–1.1	
Never Married	23,931	80.5	79.0–82.0	4.7	4.2–5.2	13.7	12.4–15.3	1.0	0.8–1.3	
Highest education level										<0.001
None/Primary school	7783	85.4	83.5–87.1	4.7	3.8–5.7	8.7	7.3–10.4	1.1	0.8–1.5	
Some Secondary school/Grade 12	22,969	82.3	80.7–83.7	4.2	3.7–4.8	12.5	11.2–13.9	1.0	0.8–1.3	
Tertiary education	3473	86.4	83.8–88.7	3.0	2.0–4.3	10.1	8.2–12.5	0.5	0.3–0.9	
Employment status										0.524
Unemployed	24,894	83.0	81.7–84.3	4.1	3.7–4.6	11.8	10.6–13.1	1.0	0.8–1.3	
Employed	8897	83.9	81.9–85.7	4.2	3.5–5.0	11.1	9.5–13.0	0.8	0.6–1.1	
Locality type										<0.001
Urban areas	20,432	86.1	84.7–87.4	4.3	3.7–4.9	8.8	7.7–10.1	0.8	0.6–1.0	
Rural informal (tribal areas)	9704	77.7	75.4–79.8	3.5	2.9–4.2	17.5	15.3–19.8	1.4	1.0–1.8	
Rural formal (farm areas)	4392	80.7	76.6–84.3	4.8	3.5–6.4	13.5	10.7–16.9	1.0	0.6–1.5	
Asset-based SES										<0.001
Low SES	18,454	79.9	77.9–81.8	4.6	4.1–5.3	14.1	12.4–16.1	1.3	1.0–1.6	
High SES	14,740	86.0	84.4–87.4	3.6	3.0–4.3	9.8	8.5–11.3	0.7	0.5–0.9	

^1^ Pearson’s chi-square test for independence *p*-values. The frequencies presented for individual variables do not necessarily sum to the total sample size (*N* = 34,528) because of missing values. The reported frequencies are based on the available observations for each variable.

**Table 4:** A bivariate multinomial logistic regression of sociodemographic factors associated with discordant results, South Africa, 2022.

Variables	HIV-Positive with Low Perceived Risk				HIV-Negative with High Perceived Risk				Self-Misdiagnosed			
	RRR	95% CI		*p*-Value	RRR	95% CI		*p*-Value	RRR	95% CI		*p*-Value
Age groups (years)												
65+	Ref				Ref				Ref			
15–24	1.63	1.08	2.46	0.021	3.02	1.75	5.22	<0.001	0.22	0.10	0.47	<0.001
25–34	3.13	2.12	4.62	<0.001	4.26	2.46	7.40	<0.001	1.24	0.66	2.33	0.512
35–44	3.93	2.60	5.94	<0.001	2.90	1.67	5.03	<0.001	1.13	0.60	2.11	0.708
45–54	3.45	2.26	5.25	<0.001	1.88	1.07	3.31	0.029	1.44	0.75	2.77	0.275
55–64	1.74	1.15	2.65	0.009	1.52	0.88	2.65	0.137	0.54	0.26	1.14	0.106
Sex												
Male	Ref				Ref				Ref			
Female	1.14	0.93	1.39	0.195	0.89	0.76	1.04	0.150	2.12	1.47	3.05	<0.001
Race groups												
Black African	Ref				Ref				Ref			
Other	0.19	0.13	0.30	<0.001	0.28	0.19	0.41	<0.001	0.19	0.08	0.46	<0.001
Marital status												
Married	Ref				Ref				Ref			
Never Married	1.67	1.35	2.06	<0.001	1.97	1.62	2.39	<0.001	1.41	0.97	2.04	0.071
Highest education level												
None/Primary school	Ref				Ref				Ref			
Some Secondary school/Grade 12	0.93	0.72	1.20	0.574	1.49	1.21	1.83	<0.001	0.94	0.67	1.32	0.715
Tertiary education	0.62	0.40	0.97	0.037	1.15	0.84	1.57	0.397	0.41	0.21	0.84	0.014
Employment status												
Unemployed	Ref				Ref				Ref			
Employed	1.00	0.81	1.23	0.981	0.93	0.77	1.12	0.454	0.75	0.52	1.09	0.130
Locality type												
Urban areas	Ref				Ref				Ref			
Rural informal (tribal areas)	0.90	0.71	1.16	0.423	2.19	1.76	2.73	<0.001	1.91	1.28	2.84	0.001
Rural formal (farm areas)	1.19	0.82	1.72	0.352	1.63	1.19	2.23	0.002	1.32	0.79	2.23	0.290
Asset-based SES												
Low SES	Ref				Ref				Ref			
High SES	0.73	0.58	0.91	0.006	0.64	0.52	0.80	<0.001	0.48	0.33	0.69	<0.001

**Table 5:** A multivariable multinomial logistic regression of sociodemographic factors associated with discordant results, South Africa, 2022.

Variables	HIV-Positive with Low Perceived Risk				HIV-Negative with High Perceived Risk				Self-Misdiagnosed			
	aRR	95% CI		*p*-Value	aRRR	95% CI		*p*-Value	aRRR	95% CI		*p*-Value
Age groups (years)												
65+	Ref				Ref				Ref			
15–24	1.04	0.60	1.79	0.901	2.11	0.90	4.93	0.085	0.11	0.04	0.35	<0.001
25–34	2.13	1.27	3.55	0.004	3.32	1.43	7.73	0.005	0.66	0.24	1.83	0.425
35–44	3.07	1.85	5.10	<0.001	2.52	1.07	5.91	0.034	0.70	0.25	1.96	0.496
45–54	2.79	1.70	4.60	<0.001	1.64	0.70	3.83	0.250	0.87	0.32	2.37	0.780
55–64	1.51	0.92	2.48	0.100	1.51	0.68	3.36	0.315	0.43	0.14	1.28	0.128
Sex												
Male	Ref				Ref				Ref			
Female	1.13	0.90	1.41	0.289	0.91	0.77	1.07	0.258	1.99	1.32	2.99	0.001
Race groups												
Black African	Ref				Ref				Ref			
Other	0.18	0.10	0.32	<0.001	0.47	0.30	0.74	<0.001	0.47	0.18	1.25	0.132
Marital status												
Married	Ref				Ref				Ref			
Never Married	1.29	0.98	1.70	0.067	1.12	0.89	1.42	0.320	2.23	1.44	3.47	<0.001
Highest education level												
None/Primary school	Ref				Ref				Ref			
Some Secondary school/Grade 12	0.87	0.64	1.17	0.359	1.29	0.98	1.69	0.068	1.25	0.76	2.06	0.377
Tertiary education	0.68	0.42	1.11	0.124	1.28	0.84	1.95	0.243	1.00	0.41	2.46	0.995
Locality type												
Urban areas	Ref				Ref				Ref			
Rural informal (tribal areas)	0.70	0.53	0.92	0.010	2.01	1.53	2.65	<0.001	1.62	0.99	2.66	0.055
Rural formal (farm areas)	1.02	0.69	1.50	0.923	1.91	1.26	2.89	0.002	1.25	0.67	2.31	0.480
Asset-based SES												
Low SES	Ref				Ref				Ref			
High SES	0.95	0.74	1.22	0.677	1.04	0.81	1.33	0.767	0.68	0.45	1.01	0.057

## Data Availability

The data used in this study are publicly available for research purposes through the Human Sciences Research Council (HSRC) data repository. The dataset can be accessed at: https://repository.hsrc.ac.za, accessed on 13 August 2026.

## References

[1] Maughan-Brown B., Venkataramani A.S. (2017). Accuracy and determinants of perceived HIV risk among young women in South Africa. BMC Public Health.

[2] Kamire V., Magut F., Khagayi S., Kambona C., Muttai H., Nganga L., Kwaro D., Joseph R.H. (2022). HIV Risk Factors and Risk Perception Among Adolescent Girls and Young Women: Results From a Population-Based Survey in Western Kenya, 2018. J. Acquir. Immune Defic. Syndr. (1988).

[3] Tugume L., Muwonge T.R., Joloba E.N., Isunju J.B., Kiweewa F.M. (2019). Perceived risk versus objectively measured risk of HIV acquisition: A cross-sectional study among HIV-negative individuals in Serodiscordant partnerships with clients attending an Urban Clinic in Uganda. BMC Public Health.

[4] Seekaew P., Pengnonyang S., Jantarapakde J., Meksena R., Sungsing T., Lujintanon S., Mingkwanrungruangkit P., Sirisakyot W., Tongmuang S., Panpet P. (2019). Discordance between self-perceived and actual risk of HIV infection among men who have sex with men and transgender women in Thailand: A cross-sectional assessment. J. Int. AIDS Soc..

[5] Machemedze T. (2023). Does self-perceived HIV risk mediate the potential association between HIV-related symbolic stigma and sexual behaviour among young adult women in Cape Town, South Africa?. BMC Public Health.

[6] Schaefer R., Thomas R., Nyamukapa C., Maswera R., Kadzura N., Gregson S. (2019). Accuracy of HIV Risk Perception in East Zimbabwe 2003–2013. AIDS Behav..

[7] Plotzker R., Seekaew P., Jantarapakde J., Pengnonyang S., Trachunthong D., Linjongrat D., Janyam S., Nakpor T., Charoenying S., Mills S. (2017). Importance of risk perception: Predictors of PrEP acceptance among Thai MSM and TG women at a community-based health service. J. Acquir. Immune Defic. Syndr. (1988).

[8] South African Government (2022). Official Guide to South Africa 2021/22 [Internet]. https://www.gcis.gov.za/official-guide-south-africa-202122.

[9] Thomas R., Galizzi M.M., Moorhouse L., Nyamukapa C., Hallett T.B. (2024). Do risk, time and prosocial preferences predict risky sexual behaviour of youths in a low-income, high-risk setting?. J. Health Econ..

[10] Pringle K., Merchant R.C., Clark M.A. (2013). Is self-perceived HIV risk congruent with reported HIV risk among traditionally lower HIV risk and prevalence adult emergency department patients? implications for HIV testing. AIDS Patient Care STDS.

[11] Mbilizi Chimwaza Y., Dadabhai S.S., Nyondo Mipando A.L., Mbeda C., Panchia R., Lucas J.P., Chege W., Hamilton E.L., Sandfort T.G.M. (2022). HIV risk perception and sexual behavior among HIV-uninfected men and transgender women who have sex with men in sub-Saharan Africa: Findings from the HPTN 075 qualitative sub-study. PLoS Glob. Public Health.

[12] Ju I., Downey L.A. (2024). The role of optimistic bias and affect on social media searches about COVID-19. Health Commun..

[13] Liu Y., Fu G., Chen Y., Wu L., Pan M., Yang Y., Chen Z., Cao Y., Li Y., Wang H. (2022). Discordance between perceived risk and actual risky sexual behaviors among undergraduate university students in mainland China: A cross-sectional study. BMC Public Health.

[14] Price J.T., Rosenberg N.E., Vansia D., Phanga T., Bhushan N.L., Maseko B., Brar S.K.M., Hosseinipour M.C., Tang J.H.M., Bekker L.-G. (2018). Predictors of HIV, HIV Risk Perception, and HIV Worry Among Adolescent Girls and Young Women in Lilongwe, Malawi. J. Acquir. Immune Defic. Syndr..

[15] Govere S.M., Galagan S., Tlou B., Mashamba-Thompson T., Bassett I.V., Drain P.K. (2021). Effect of perceived HIV risk on initiation of antiretroviral therapy during the universal test and treat era in South Africa. BMC Infect. Dis..

[16] Zuma K., Zungu N.P., Moyo S., Marinda E., Simbayi L.C., Jooste S.E., Mabaso M., Ramlagan S., Makola L., van Zyl J. (2025). The Sixth South Africa National HIV, Incidence and Behaviour Survey, 2022: A Summary Report.

[17] Mabaso M., Makola L., Naidoo I., Mlangeni L.L., Jooste S., Simbayi L. (2019). HIV prevalence in South Africa through gender and racial lenses: Results from the 2012 population-based national household survey. Int. J. Equity Health.

[18] Human Sciences Research Council (HSRC) (2018). The Fifth South African National HIV Prevalence, Incidence, Behaviour and Communication Survey, 2017 (SABSSM V1).

[19] Bell G.J., Ncayiyana J., Sholomon A., Goel V., Zuma K., Emch M. (2022). Race, place, and HIV: The legacies of apartheid and racist policy in South Africa. Soc. Sci. Med..

[20] Fagbamigbe A.F., Adebayo S.B., Idemudia E. (2016). Marital status and HIV prevalence among women in Nigeria: Ingredients for evidence-based programming. Int. J. Infect. Dis..

[21] Shisana O., Zungu-Dirwayi N., Toefy Y., Simbayi L.C., Malik S., Zuma K. (2004). Marital status and risk of HIV infection in South Africa. S. Afr. Med. J..

[22] Legarth R., Omland L.H., Kronborg G., Larsen C.S., Pedersen C., Gerstoft J., Obel N. (2014). Educational attainment and risk of HIV infection, response to antiretroviral treatment, and mortality in HIV-infected patients. AIDS.

[23] Kayeyi N., Sandøy I.F., Fylkesnes K. (2009). Effects of neighbourhood-level educational attainment on HIV prevalence among young women in Zambia. BMC Public Health.

[24] Mabaso M., Sokhela Z., Mohlabane N., Chibi B., Zuma K., Simbayi L. (2018). Determinants of HIV infection among adolescent girls and young women aged 15–24 years in South Africa: A 2012 population-based national household survey. BMC Public Health.

[25] Maulsby C.H., Ratnayake A., Hesson D., Mugavero M.J., Latkin C.A. (2020). A Scoping Review of Employment and HIV. AIDS Behav..

[26] Bunyasi E.W., Coetzee D.J. (2017). Relationship between socioeconomic status and HIV infection: Findings from a survey in the Free State and Western Cape Provinces of South Africa. BMJ Open.

[27] Wabiri N., Taffa N. (2013). Socio-economic inequality and HIV in South Africa. BMC Public Health.

[28] Gibbs A., Reddy T., Dunkle K., Jewkes R. (2020). HIV-Prevalence in South Africa by settlement type: A repeat population-based cross-sectional analysis of men and women. PLoS ONE.

[29] Iannotta J.G., Nightingale E.O., Fischhoff B. (2001). Adolescent Risk and Vulnerability: Concepts and Measurement.

[30] Steinberg L. (2008). A social neuroscience perspective on adolescent risk-taking. Dev. Rev..

[31] Elakpa D., Chamane S., Dlamini S.S., Hlubi F.L., Olusanya T.O., Masuku M.M. (2025). Factors Associated with Sexual Behaviour among Women Aged 15–49 in South African Low-Income Communities. McGill J. Glob. Health.

[32] Berhan Y., Berhan A. (2015). A meta-analysis of risky sexual behaviour among male youth in developing countries. AIDS Res. Treat..

[33] UNAIDS (2019). UNAIDS 2019 Data.

[34] Chory A., Gillette E., Callen G., Wachira J., Sam-Agudu N.A., Bond K., Vreeman R. (2023). Gender differences in HIV knowledge among adolescents and young people in low-and middle-income countries: A systematic review. Front. Reprod. Health.

[35] Senderowicz L., Bullington B.W., Sawadogo N., Tumlinson K., Langer A., Soura A., Zabré P., Sié A. (2023). Measuring Contraceptive Autonomy at Two Sites in Burkina Faso: A First Attempt to Measure a Novel Family Planning Indicator. Stud. Fam. Plan..

[36] Some S.Y.M., Pu C., Huang S.L. (2021). Empowerment and use of modern contraceptive methods among married women in Burkina Faso: A multilevel analysis. BMC Public Health.

[37] Kriel Y., Milford C., Cordero J., Suleman F., Beksinska M., Steyn P., Smit J.A. (2019). Male partner influence on family planning and contraceptive use: Perspectives from community members and healthcare providers in KwaZulu-Natal, South Africa. Reprod. Health.

[38] Nyirenda M., Mnqonywa N., Tutshana B., Naidoo J., Kowal P., Negin J. (2022). An analysis of the relationship between HIV risk self-perception with sexual behaviour and HIV status in South African older adults. Afr. J. AIDS Res..

[39] McGrath N., Hosegood V., Newell M.L., Eaton J.W. (2015). Migration, sexual behaviour, and HIV risk: A general population cohort in rural South Africa. Lancet HIV.

[40] Madiba S., Ngwenya N. (2017). Cultural practices, gender inequality and inconsistent condom use increase vulnerability to HIV infection: Narratives from married and cohabiting women in rural communities in Mpumalanga province, South Africa. Glob. Health Action.

[41] Chasimpha S.J.D., McLean E.M., Dube A., McCormack V., Dos-Santos-Silva I., Glynn J.R. (2020). Assessing the validity of and factors that influence accurate self-reporting of HIV status after testing: A population-based study. AIDS.

[42] Nuvunga S., Langa D.C., Sacarlal J., Rossetto E., Baltazar C.S. (2024). HIV prevalence and associated factors among married women, Mozambique, 2015: Analysis of the 2015 National AIDS Indicator Survey (IMASIDA). Pan Afr. Med. J..

[43] Smit R. (2008). HIV and AIDS: Are all women equally at risk? Afrikaans speaking married women’s perceptions of self-risk. J. Comp. Fam. Stud..

[44] Afriyie J., Essilfie M.E. (2019). Association between risky sexual behaviour and HIV risk perception among in-school adolescents in a municipality in Ghana. Ghana Med. J..

[45] Buthelezi M.M., Zulu Z.P., Dick S. (2024). The impacts of social exclusion and inequality in rural communities: A qualitative study in Limpopo Province, South Africa. Int. J. Dev. Sustain..

[46] Schatz E., Houle B., Mojola S.A., Angotti N., Williams J. (2019). How to “Live a Good Life”: Aging and HIV Testing in Rural South Africa. J. Aging Health.

[47] Mahlalela N.B., Manne-Goehler J., Ohene-Kwofie D., Adams L.B., Montana L., Kahn K., Rohr J.K., Bärnighausen T., Gómez-Olivé F.X. (2024). The Association Between HIV-Related Stigma and the Uptake of HIV Testing and ART Among Older Adults in Rural South Africa: Findings from the HAALSI Cohort Study. AIDS Behav..

